# Pre-Hydration and a Forced Diuresis Protocol for ^18^F-FDG PET/CT Yielded an Optimal Effect on Primary Pelvic Malignancies

**DOI:** 10.3390/jcm13206090

**Published:** 2024-10-12

**Authors:** Yi-Chia Hsieh, Wei-Jen Yao, Nan-Tsing Chiu, Wen-Horng Yang, Ho-Shiang Huang

**Affiliations:** 1 Departments of Urology, National Cheng Kung University Hospital, College of Medicine, National Cheng Kung University, Tainan 704302, Taiwanwhyang@mail.ncku.edu.tw (W.-H.Y.); 2 Departments of Nuclear Medicine, Chia-Yi Christian Hospital, Chiayi 600566, Taiwan; wjyao@mail.ncku.edu.tw; 3 Departments of Nuclear Medicine, National Cheng Kung University Hospital, College of Medicine, National Cheng Kung University, Tainan 704302, Taiwan; ntchiu@mail.ncku.edu.tw

**Keywords:** pelvic oncology, ^18^F-fluorodeoxyglucose, hydration, PET/CT, forced diuresis

## Abstract

**Background:** Positron emission tomography (PET) with ^18^F-FDG is being used more frequently to evaluate primary pelvic tumors (PTs). However, a standardized hydration protocol is essential for an optimal diuretic effect and constant results. **Methods:** We reviewed 109 patients with PTs who had undergone ^18^F-FDG PET/CT examinations between November 2006 and April 2013. Four different protocols were used: (a) no hydration (group 1); (b) oral hydration (800 mL) after an early scan (group 2); (c) intravenous (IV) hydration (500 mL) during an early scan followed by oral hydration (800 mL) and IV furosemide (20 mg) after an early scan (group 3); and (d) oral hydration (800 mL) before an FDG injection followed by the protocol from group 3 (group 4). The maximum standardized uptake (SUV_max_) of the urinary bladder (UB) and PTs and the PT/UB SUV_max_ ratios were examined. **Results:** The UB SUV_max_ of group 4 was significantly lower in the early scan compared to that in the other three groups. Group 4 had a significantly higher PT/UB SUV_max_ ratio in the early scan than the other three groups, and it also had a 52.5% positivity rate for PTs. **Conclusions:** The pre-hydration plus forced diuresis protocol yielded the optimal effect of UB radiotracer washout and had the best PT/UB SUV_max_ ratio in both scans.

## 1. Introduction

Accurate staging and detection of metastases in pelvic tumors (PTs) are essential components in regard to the choice of appropriate treatment and the avoidance of radical surgery in incurable patients. ^18^F-FDG PET/CT is a currently recommended noninvasive tool for detecting and localizing unknown primary tumors, differentiating malignant from benign tissue, staging and evaluating recurrence, differentiating recurrence from postsurgical changes, and monitoring the response to therapy [1,2]. This underscores its pivotal role in comprehensive patient management and highlights its utility across multiple aspects of pelvic tumor evaluation and treatment planning.

However, the urinary excretion of ^18^F-FDG through the kidneys into the urinary tract hampers the evaluation of PTs, particularly in urinary bladder (UB) cancer [3,4]. This might be why ^18^F-FDG (F-fluorodeoxyglucose) PET/CT has not been useful in local staging in previous studies but might currently have a role in detecting nodal and distant metastases [4,5]. The major source of uncertainty in the interpretation of ^18^F-FDG PET/CT images is the anatomic position of PTs that are contiguous to the UB [6]. Because of the overlap between FDG activity at tumor sites and physiological radioactivity in urine, the role of ^18^F-FDG PET/CT in detecting primary PTs is limited. Hence, patient preparation is important for reducing tracer uptake in healthy tissue and for maintaining and optimizing tracer uptake in the target structure [7,8].

The procedural guidelines of the European Association of Nuclear Medicine (EANM) [8] suggest that drinking one liter of water during the 2 h before an FDG injection is important for ensuring a sufficiently low concentration of FDG in the urine and for radiation safety. Some protocols have been reported to improve PET/CT scanning by defining many confounding ^18^F-FDG-avid lesions that stem from the lower urinary tract or its neighborhood, such as delayed images after diuretic and oral hydration [9,10,11,12] or forced diuresis coupled with parenteral hydration [13]. Five studies [9,10,11,12,13] reported lower radiotracer activity in the UB and improved visualization of lower pelvic structures after patients had been treated with intravenous (IV) furosemide (Lasix) and different amounts of oral fluid intake (500 mL, 500–750 mL, 800–1000 mL, 1500–2000 mL, and 500–1000 mL). However, they did not focus on pelvic tumors and did not clarify why they chose the amount of fluid for each patient, nor did they state whether their hydration protocols resulted in an equal hydration status for each patient before the FDG injection, whether they had an optimal diuretic effect, or whether they resulted in a constant result.

The Society of Nuclear Medicine and Molecular Imaging mentioned only oral water hydration before FDG injection, but no more suggestions regarding the amount of post-injection hydration and post-injection diuretic usage were made. While the EANM guidelines advocated for oral pre-hydration, subsequent research has delved into the impact of both the volume and timing of hydration to further refine ^18^F-FDG PET/CT diagnostic accuracy. Different modifications of hydration protocols via oral or parenteral hydration combined with intravenous (IV) furosemide yielded different pelvic malignancy detection rates [8,14,15]. Recognizing that clinical guidelines must evolve in response to emerging evidence, we assert that evaluating the presence or absence of oral pre-hydration across different patient groups remains a pertinent area of investigation. This is particularly true for patients with pelvic tumors, who are at higher risk of experiencing obscured imaging effects. Our objective was to share our experience of establishing a standardized and optimized hydration protocol to enhance positivity rates for pelvic tumors in PET/CT scans. We conducted a comparative analysis among patients with pelvic tumors, evaluating four distinct hydration protocols with and without furosemide treatment to assess their impact on PET/CT image quality. Through this investigation, we aimed to identify the best practices and to refine protocols to improve diagnostic accuracy and therapeutic management in patients with pelvic tumors undergoing PET/CT imaging.

## 2. Materials and Methods

From November 2006 to April 2013, a comprehensive investigation was undertaken involving 109 patients harboring various primary tumors (PTs) encompassing 74 cases of cervical cancers, 22 UB (urinary bladder) cancers, 4 instances of vaginal cancers, 2 ovarian cancers, and 7 occurrences of colon cancers. These patients were carefully selected to participate in staging or restaging procedures utilizing whole-body 18F-FDG PET/CT at the esteemed Department of Nuclear Medicine within the premises of the National Cheng Kung University Hospital (NCKUH). All patients were fully informed about the procedures. The study, bearing the identification code B-ER-104-331, received formal endorsement from the NCKUH Institutional Review Board on 21 December 2015, underscoring its compliance with ethical standards and guidelines. Each potential participant was duly furnished with informed written consent, reflecting their voluntary engagement in the study.

Exhaustive efforts were made to ascertain the eligibility of participants, with exclusion criteria meticulously outlined to exclude individuals with a history of previous pelvic surgery, radiotherapy, systemic chemotherapy, chronic kidney disease at stage 5 [16], and inflammatory diseases such as Crohn’s disease, retroperitoneal mycobacterium, and IgG4-related disease. Every patient underwent standard staging procedures and surgical treatment depending on the local stage of the PT. The validity and reliability of the PET/CT findings were upheld through a rigorous validation process, leveraging a reference standard consisting of either histopathological examinations or comprehensive clinical and radiological follow-ups spanning a duration of 12 months. This meticulous approach aimed to ensure the accuracy and integrity of the study’s findings, thereby contributing to advancements in the understanding and management of PTs.

### 2.1. FDG–PET Imaging

**Figure 1:**
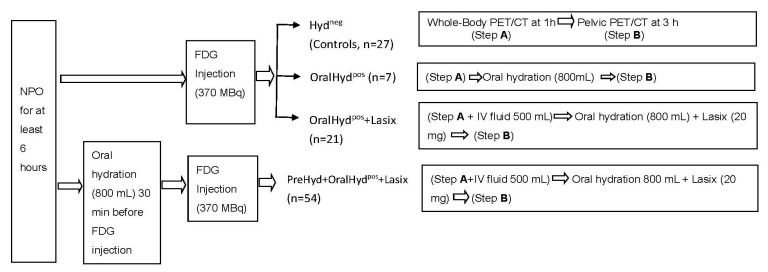
The four different hydration protocols that were designed for this study.

Whole-body FDG–PET scans were performed (Biograph 6 scanner; Siemens Medical Solutions, Erlangen, Germany) on all referred patients to stage their primary PTs with or without associated lymph node metastases. All patients fasted from midnight until 9 A.M. the next morning, when they were injected (IV) with 370 MBq of ^18^F-FDG and had their glucose levels, which had to be less than 130 mg/dL, measured before scanning. Sixty minutes later, a whole-body scan (early scan) with an effective field of view extending from the head to the upper thighs was performed. About 3 h after the FDG injection, an additional scan (delayed scan) was performed. Four different hydration protocols were used consecutively (Figure 1), with a different one being utilized for each group: (a) group 1: no hydration (controls: 27 patients); (b) group 2: oral hydration (800 mL) after an early scan (7 patients) [17]; (c) group 3: IV hydration (500 mL) during an early scan followed by oral hydration (800 mL) and IV furosemide (Lasix) (20 mg) after an early scan (21 patients) [12]; and (d) group 4: oral hydration (800 mL) before an FDG injection followed by IV hydration (500 mL) during an early scan, followed by oral hydration (800 mL) and IV furosemide after the early scan (54 patients) [18]. A delayed scan was acquired 3 h after the FDG injection. Patients were instructed to void frequently. The patients in group 4 had 3 voidings on average before image acquisition, and each whole-body PET/CT scan was performed when the UB was filled for the fourth time. After completing the examination, every patient was required to undergo a thorough two-hour observation period within the examination room. This precautionary measure was undertaken to meticulously ascertain and ensure the absence of any potential side effects or adverse reactions.

### 2.2. Image Evaluation

All PET/CT images were interpreted by 2 experienced nuclear medicine physicians who knew all the clinically relevant information about each patient. A cross-calibration process was regularly performed to ensure the stability of measurements between different scanners. To compare the effects of the different hydration protocols on the positivity rate for primary PTs, an in-depth analysis was performed. We examined the changes in the variables (the UB, PTs, and PT/UB SUV_max_ ratios of the four groups) between early and delayed PET scans with the ^18^F-FDG concentrations.

### 2.3. Statistics

For comprehensive statistical analyses, SPSS 16.0 for Windows (SPSS Inc., Chicago, IL, USA) served as the primary analytical tool. The data were meticulously presented as mean ± standard deviation (SD). Variations in SUVmax changes between groups were rigorously scrutinized using a combination of analytical methods, including the Mann–Whitney U test, Fisher’s exact test, and one-way ANOVA (analysis of variance), supplemented with subsequent post hoc tests as deemed appropriate. A predetermined threshold of significance was established at *p* < 0.05, ensuring robustness and reliability in the interpretation of findings.

## 3. Results

We enrolled 109 patients (mean age: 55.6 years; age range: 27–83 years) (Table 1). There were no significant differences in age in the four groups. This study included 90 female and 19 male participants as subjects. In the event of inefficacy, our protocol was promptly adjusted, with the previous one being discontinued, and the subsequent patients transitioned to the next protocol in the sequence. This process involves the utilization of four protocols systematically categorized into four sequential groups. Such adaptive measures ensure the optimization of treatment strategies, facilitating a comprehensive exploration of various approaches to address the challenges encountered during the course of patient care.

**Figure 2:**
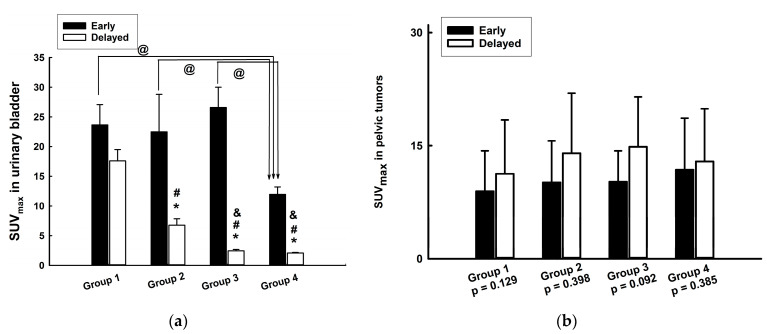
Results for the maximum standardized uptake value (SUV_max_) in (**a**) the urinary bladder (UB) and in (**b**) a pelvic tumor (PT) 1 h (early) and 3 h (delayed) after an ^18^F-FDG injection in the four different groups. * *p* < 0.05 compared with the early scan in the same group; # *p* < 0.05 compared with the delayed scan in group 1; @ *p* < 0.05 compared with the early scans in group 4; ^&^ *p* < 0.05 compared with the delayed scan in group 2.

The UB SUV_max_ of the three hydrated groups (groups 2 (*p* = 0.039), 3 (*p* < 0.05), and 4 (*p* < 0.05)) was significantly lower in the delayed scan than in the early scan (Figure 2a). The ANOVA with post hoc multiple comparisons showed significant differences in the SUV_max_ of the UB between groups 1 and 4 (*p* = 0.003), 2 and 4 (*p* = 0.044), and 3 and 4 (*p* < 0.001) in the early scan (Figure 2a). In the delayed scan, the SUV_max_ of the UB in all three hydrated groups (i.e., groups 2, 3, and 4) was significantly (*p* < 0.05) lower than that in group 1. The SUV_max_ of the UB in the delayed scan was also significantly lower in groups 3 (*p* = 0.002) and 4 (*p* < 0.001) than that in group 2.

The SUV_max_ of PTs had no significant differences between the delayed and the early scans in each group (Figure 2b). There were no significant differences in the PT SUV_max_ among the four groups in the early or delayed scan.

**Figure 3:**
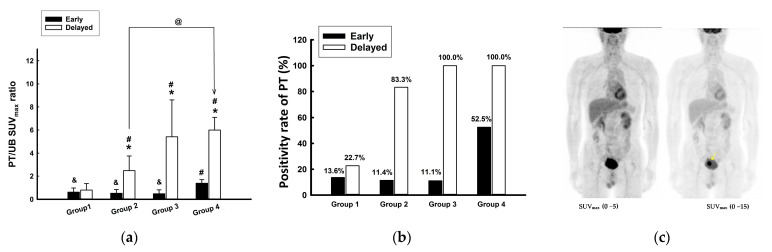
(**a**) Changes in the pelvic tumor/urinary bladder (PT/UB) SUV_max_ (maximum standardized uptake value) ratios in different groups in the early and delayed scans. (**b**) The positivity rate of PTs in different scan modes in each group. (**c**) By adjusting the grayscale, bladder cancer (arrow) could be clearly identified in the early scan of group 4. * *p* < 0.05 compared with the early scan in the same group; # *p* < 0.05 compared with the control group in each early and delayed scan; @ *p* < 0.05 compared with the delayed scan in group 2; ^&^ *p* < 0.05 compared with the early scan in group 4.

There were no significant differences in the PT/UB SUV_max_ ratios between the early and delayed scans in group 1; however, there were significant differences before and after hydration in groups 2 (*p* = 0.018), 3 (*p* < 0.001), and 4 (*p* < 0.001) (Figure 3a). Group 4 had a significantly (*p* = 0.008) higher PT/UB SUV_max_ ratio in the early scan than groups 1, 2, and 3, whereas, in the delayed scan, the PT/UB SUV_max_ ratios in all hydration groups (i.e., groups 2, 3, and 4) were significantly higher than that in group 1 (*p* < 0.05). Furthermore, the PT/UB SUV_max_ ratio in the delayed scan of group 4 was significantly higher than that of group 2. The positivity rate (defined as PT SUV_max_/UB SUV_max_ > 1) of the PTs in the early scan was higher in group 4 (mean = 52.5%; cervical cancer (CC) = 37.0%; UB cancer (UBC) = 100%) than in groups 1 (13.6%), 2 (11.4%), and 3 (11.1%) (Figure 3b). Through adjusting the gray scale, bladder cancer (pointed out by the arrow) became clearly discernible during the initial scan of group 4 (Figure 3c). The PT positivity rate in the delayed scan was higher in groups 2 (83.3%), 3 (100%), and 4 (100%) than in group 1 (22.7%).

**Figure 4:**
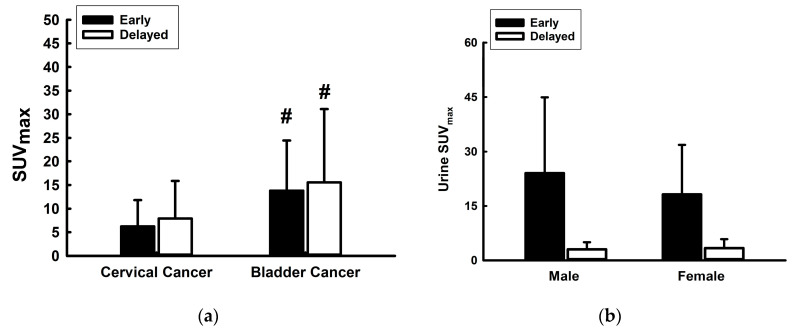
Changes in the tumor SUV_max_ (maximum standardized uptake value) in the early and delayed scans of (**a**) bladder cancer (in all four groups) and cervical cancer (in all four groups) and in (**b**) the urine SUV_max_ in males and females. # *p* < 0.05 compared with tumor SUV_max_ between cervical cancer and bladder cancer in the early and delayed scans.

No notable disparities were observed in the maximum standardized uptake value (SUVmax) of primary tumors (PTs) between the early and delayed scans among patients with cervical cancer (CC) or urinary bladder cancer (UBC) (Figure 4a); however, SUV_max_ was significantly higher in the early (*p* = 0.025) and delayed (*p* < 0.001) scans of patients with UBC than in those with CC. Gender-based variations in the SUVmax of urine during both early and delayed scans were negligible (Figure 4b). These findings underscore the distinct metabolic characteristics inherent to different types of malignancies, particularly evident in the context of urinary bladder cancer.

## 4. Discussion

^18^F-FDG, which is radioactive, allows for the evaluation of glucose metabolism in healthy cells and in tumor cells. Unlike glucose, however, ^18^F-FDG is not completely reabsorbed in the proximal tubules of the kidney but is excreted with the urine [19]. Hence, the physiological accumulation of ^18^F-FDG can interfere with the diagnostic evaluation of the pelvic regions even if the UB is emptied before the scan [19,20]. Another factor that might interfere with the quality of a PET/CT examination is the hydration level of the human body, because an adequate hydration level reduces radiotracer uptake in healthy tissue and keeps a patient’s radiation exposure levels as low as possible [7,8]. Moran et al. [21] reported that the total amount of ^18^F-FDG excretion is related to the hydration level. The effect of adequate hydration was thought to lower the radioactivity in healthy tissue and to increase the target-to-background ratio [14,22]. Protocols with a diuretic and oral hydration (500–1000 mL) after an FDG injection [9,10,11,12], as well as with forced diuresis plus IV hydration (500 mL of normal saline) [13], lowered tracer activity in the UB and improved the visualization of lower PTs. However, the effect of combining pre-hydration and forced diuresis after an FDG injection has not yet been evaluated.

Applying the same hydration protocol after an FDG injection to each patient yielded different PT/UB SUV_max_ ratios with large variations within the same group. This implied that the hydration levels varied in the examined patients. Comorbidities and 6–9 h of fasting might have been the main causes for these variations. In fact, there are three types of dehydration: isotonic, hypotonic, and hypertonic [23]. Dehydration might be exacerbated by comorbidities, polypharmacy, and physical and mental disabilities [23,24]. Therefore, hydrating patients before and after an FDG injection can equalize the baseline hydration levels before a PET/CT examination. A dual-phase PET/CT technique with forced diuresis and oral hydration [25] suggested that an early scan detected distant metastasis and that a delayed post-diuretic scan helped assess primary PTs and the perivesical/pelvic area for regional nodal metastasis. Therefore, oral hydration before FDG injection plus forced diuresis (group 4’s hydration protocol) provides at least three advantages: one is that it improves the PT/UB SUV_max_ ratio in the early scan with a higher positivity rate, the second is that oral hydration is the simplest and least expensive method, and the third is the constant SUV_max_ results with less variation in the same pelvic cancer group.

In our hospital, due to administrative factors, patients scheduled to undergo the FDG PET/CT study were uniformly restricted from eating after midnight and were scheduled for examinations starting at 9 A.M. on the following day; therefore, every patient needed to have a 9 h fasting period before a PET/CT examination. According to Hodgkinson’s (2003) standard for the recommended daily fluid intake, a 70 kg patient needs at least 2100–2250 mL per day. Due to the uneven hydration status of each patient during the nine-hour period before the examination, 787.5–843.8 mL of water supplementation for adequate hydration before an FDG PET/CT study was performed [22]. Therefore, the patients in group 4 were orally hydrated with 800 mL before FDG injection for the equalization of fluid status.

We found that the best hydration protocol was oral hydration (800 mL) before an FDG injection and IV hydration (500 mL) during an early scan followed by oral hydration (800 mL) and IV furosemide after an early scan (group 4). Oral hydration with 800 mL before the FDG injection significantly lowered the urinary SUV_max_ in the early scan and significantly raised the PT/UB SUV_max_ ratio in the early scan compared with those in groups 1, 2, and 3; in addition, it significantly increased the PT/UB SUV_max_ ratio in the delayed scan compared with those in groups 1 and 2. It also provided the following benefits: 100% and 52.5% positivity rates for UBCs and for other examined PTs in the PET/CT early scans, respectively.

The SUV_max_ was used to quantify FDG uptake. It is well known that aggressive tumors tend to have higher levels of FDG uptake than those of less aggressive tumors [26,27]. In a large oncology practice, high tumor FDG uptake predicts a worse prognosis [28,29,30]. Bladder transitional cell carcinoma is FDG-avid [31]. In the present study, we found that the SUV_max_ of patients with UBC was higher than that of patients with CC in early and in delayed scans. The hydration protocol in group 4 increased the primary pelvic tumor positivity rate even in the early scan (Figure 3b), and this effect was more significant in patients with UBC than in those with CC. However, UBC is not on the U.S. National FDG PET Coverage List, but other pelvic cancers, such as colorectal cancer, ovarian cancer, and CC, are included. We found that an FGD PET/CT scan was a good choice for the initial treatment strategy for UBC. In fact, the NCCN guidelines^®^ for UBC suggest that a PET/CT scan might be beneficial in staging and follow-up for selected patients with T2 disease (muscle-invasive disease) and in patients with ≥cT3 disease.

No side effects were noticed during the whole procedure. All patients received close observation in our examination room for 2 h after the examination. According to the previous literature, in male patients, urine retention might occur after forced diuresis, especially in older patients with benign prostate hypertrophy. One study suggested that three successive voidings of the urinary bladder were sufficient to bring urinary activity to a background level. Our hydration protocol for group 4, which involved instructing patients to void at least three times before we gave them PET/CT scans, effectively prevented urine retention. There were no dizziness, falls, hypotension, or other bothersome complaints in our experiment. Since our research prioritized patient-centric care and aimed to deliver the best diagnostic outcomes for patients, upon realizing that the effectiveness of group 2 was not particularly promising, we transitioned the remaining patients to alternative protocols. Among these, that of group 4 demonstrated the most favorable outcomes. Therefore, to ensure the best diagnostic effect for patients, all subsequent patients were placed on the fourth protocol. This uneven distribution of participants among groups was a clinical decision aimed at optimizing patients’ clinical outcomes.

Due to the minimal side effects and better positivity rate for pelvic tumors of the protocol involving pre-hydration plus forced diuresis, this approach can be promoted for clinical applications. We also found that our pre-hydration plus forced diuresis protocol (group 4) could be used for malignancies of the upper urinary tract, but this finding needs to be confirmed through additional clinical studies. The limitations of our study primarily stem from two key factors. First, there is a selection bias due to the heterogeneity of pelvic cancer types across the four groups, which restricts the generalizability of the findings. Future studies will aim to address this by ensuring a more balanced representation of pelvic tumor types in each subgroup. This will enhance the robustness of the results and enable more tailored therapeutic approaches for pelvic malignancies. Second, the lack of oral pre-hydration in groups 1 to 3, which would have allowed for a more meaningful comparison, presents a flaw in the study design. Although this limitation is inherent to the retrospective nature of the research, future prospective studies will prioritize the standardization of pre-hydration protocols to facilitate clearer and more comparable outcomes. Our findings contribute meaningful insights, particularly regarding the timing and volume of hydration. Importantly, our study focuses specifically on patients with pelvic tumors, providing a unique perspective not covered in other studies. This focus underscores the continued relevance of our research in informing best practices for PET/CT imaging in this patient population. Overall, the promising outcomes observed with the pre-hydration plus forced diuresis protocol underscore its potential to significantly advance diagnostic and therapeutic strategies for various types of pelvic cancers, paving the way for improved patient outcomes and quality of care in clinical practice.

## 5. Conclusions

The pre-hydration combined with forced diuresis protocol (group 4) demonstrated superior outcomes in terms of urinary bladder (UB) radiotracer washout and the detection of primary tumors (PTs) in both early and delayed scans. Remarkably, both male and female subjects exhibited excellent tolerance to this protocol, experiencing no adverse effects. Notably, the maximum standardized uptake value (SUVmax) of primary urinary bladder cancer (UBC) was significantly elevated compared to cervical cancer (CC).

By employing our pre-hydration plus forced diuresis protocol, the future utility and efficacy of PET/CT scans in clinical settings, particularly for pelvic tumors such as bladder cancer, are poised for substantial enhancement. While further studies are needed to validate these findings, this protocol shows promise for enhancing diagnostic accuracy and therapeutic management in patients with pelvic malignancies. These early results suggest that it may contribute meaningfully to future clinical practice, though additional research will be essential to confirm its broader applicability.

## Figures and Tables

**Table 1:** Patient characteristics by group.

	Group 1	Group 2	Group 3	Group 4
No. of cases	*n* = 27	*n* = 7	*n* = 21	*n* = 54
Gender (Male/Female)	0/27	2/5	9/12	8/46
Age (years)	56.2 ± 14.8	55.8 ± 7.7	58.6 ± 10.8	53.9 ± 12.6
Cancer type (*n*)				
Cervical	26	4	7	37
Vaginal	1	0	0	3
Ovarian	0	1	0	1
Bladder	0	2	7	13
Others	0	0	7	0

Data are the mean ± standard deviation. Group 1, controls: not hydrated. Group 2, orally hydrated (800 mL) and IV hydration (500 mL) after an early scan. Group 3, orally hydrated (800 mL) and IV hydration (500 mL) plus intravenous (IV) furosemide (Lasix) (20 mg) injection after an early scan. Group 4, orally hydrated (800 mL) before an FDG injection followed by oral hydration (800 mL) and IV hydration (500 mL) plus IV furosemide (20 mg) after an early scan.

## Data Availability

The data presented in this study are available upon request from the corresponding author. The data are not publicly available due to ethical restrictions.
